# Comparative Analysis of Microleakage of Zirconia-infused Glass Ionomer Cement with Miracle Mix and Amalgam: An In Vitro Study

**DOI:** 10.7759/cureus.3672

**Published:** 2018-12-03

**Authors:** Eeraveni Ranadheer, Utsav D Shah, Kiran Kumar Neelakantappa, Shoba Fernandes

**Affiliations:** 1 Paediatric & Preventive Dentistry, Mithila Minority Dental College & Hospital, Bihar, IND; 2 Paediatric & Preventive Dentistry, Utsav Paediatric & Multispeciality Dental Clinic, Surat, IND; 3 Endodontics, Government Dental College and Research Institute, Bangalore, IND; 4 Paediatric & Preventive Dentistry, Narsinhbhai Patel Dental College & Hospital, Ahmedabad, IND

**Keywords:** dye penetration, microleakage, zirconomer

## Abstract

Context

Various restorative materials are introduced in dentistry to achieve adequate strength and restore aesthetics. Dental amalgam is a versatile material with self-sealing property, but is unaesthetic. Other restorative materials like composites require conservative preparation, but exhibit polymerisation shrinkage resulting in microleakage. To overcome these drawbacks, a high strength restorative material reinforced with ceramic and zirconia fillers known as Zirconomer (Shofu Inc., Japan) has been introduced.

Aims

This in vitro study aims to evaluate and compare the microleakage of zirconia-infused glass ionomer cement (Zirconomer) with Miracle Mix (GC Fuji Miracle Mix, Japan) and amalgam.

Materials and methods

In this in vitro study, 30 non-carious premolar teeth were randomly divided into three groups (n=10) depending on the restorative material used—Silver Amalgam (DPI, India), Miracle Mix, and Zirconomer. Standard Class V cavities were prepared on the buccal surface of 30 non-carious extracted premolars. The restored teeth were thermocycled and then immersed in 2% methylene blue dye for 24 hours. All teeth were bisected longitudinally in a buccolingual direction and observed under a stereo microscope at 40X magnification for the evidence of dye penetration. The data were analyzed using one-way analysis of variance (ANOVA) and Kruskal-Walis tests (p<0.01).

Results

Zirconomer showed the least microleakage in Class V cavity restoration with a statistically significant difference to amalgam and Miracle Mix.

Conclusions

Zirconomer has proven to be an excellent restorative material as it showed the least microleakage followed by Miracle Mix and amalgam. Zirconomer raises the bar for restorative reinforced glass ionomers by outperforming conventional glass ionomers and amalgam restoration.

## Introduction

Restorative dentistry has experienced an upheaval to reinstitute teeth to normal form and function. In this dental epoch, conservation of tooth structure is of utmost importance [1]. The dental material should attain the original form of the tooth and secure the cavity [2]. Microleakage is defined as the clinically detectable passage of bacteria, fluids, molecules or ions between the restorative material and the cavity wall it is applied to [1]. Secondary caries and pulpal irritation are common consequences of microleakage resulting in the failure of restoration [3].

Dental amalgam has been the material of choice in restorative dentistry for 170 years. Microleakage develops due to the absence of bonding between the amalgam and the tooth structure [4]. The monopoly of dental amalgam was replaced by other enviable materials [1]. Simmons in 1977 proposed the addition of amalgam alloy to glass ionomer cement (GIC) to improve strength and impart radiopacity. Poor aesthetics and the failure to take burnish limited their use. The absence of interfacial bonding foreshortened their wear resistance [5,6].

A new generation of GICs—Zirconomer (Shofu Inc., Japan)—was developed by incorporating particles of zirconia in order to achieve greater compressive and flexure strengths, as well to attain less occlusal wear and fast setting reaction [7]. Studies pertaining to the comparative evaluation of the microleakage of Zirconomer are sparse in literature.

The present study, therefore, aims to evaluate and compare the microleakage of Silver Amalgam (DPI, India), Miracle Mix (GC Fuji Miracle Mix, Japan), and zirconia-infused glass ionomer cement, Zirconomer.

## Materials and methods

The present in vitro study was carried out in the Department of Paediatric & Preventive Dentistry, Dental College and Hospital. Thirty non-carious human extracted premolar teeth were collected, subjected to ultrasonic cleaning, and stored in 0.2% thymol solution at room temperature until used as test specimens [8].

The teeth were randomly assigned to three groups of 10 each.

Group I

**Figure 1:**
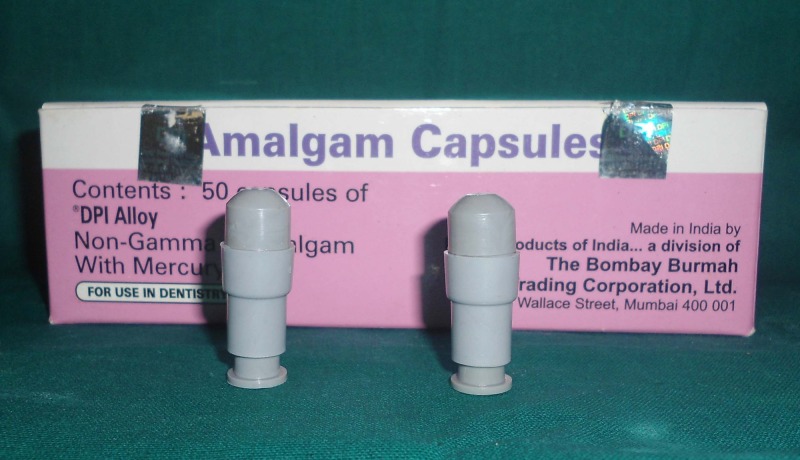
Dental amalgam (DPI, Mumbai, India)

Teeth restored with amalgam [DPI, Mumbai, India. Batch No – 3141 (Figure 1)]

Group II

**Figure 2:**
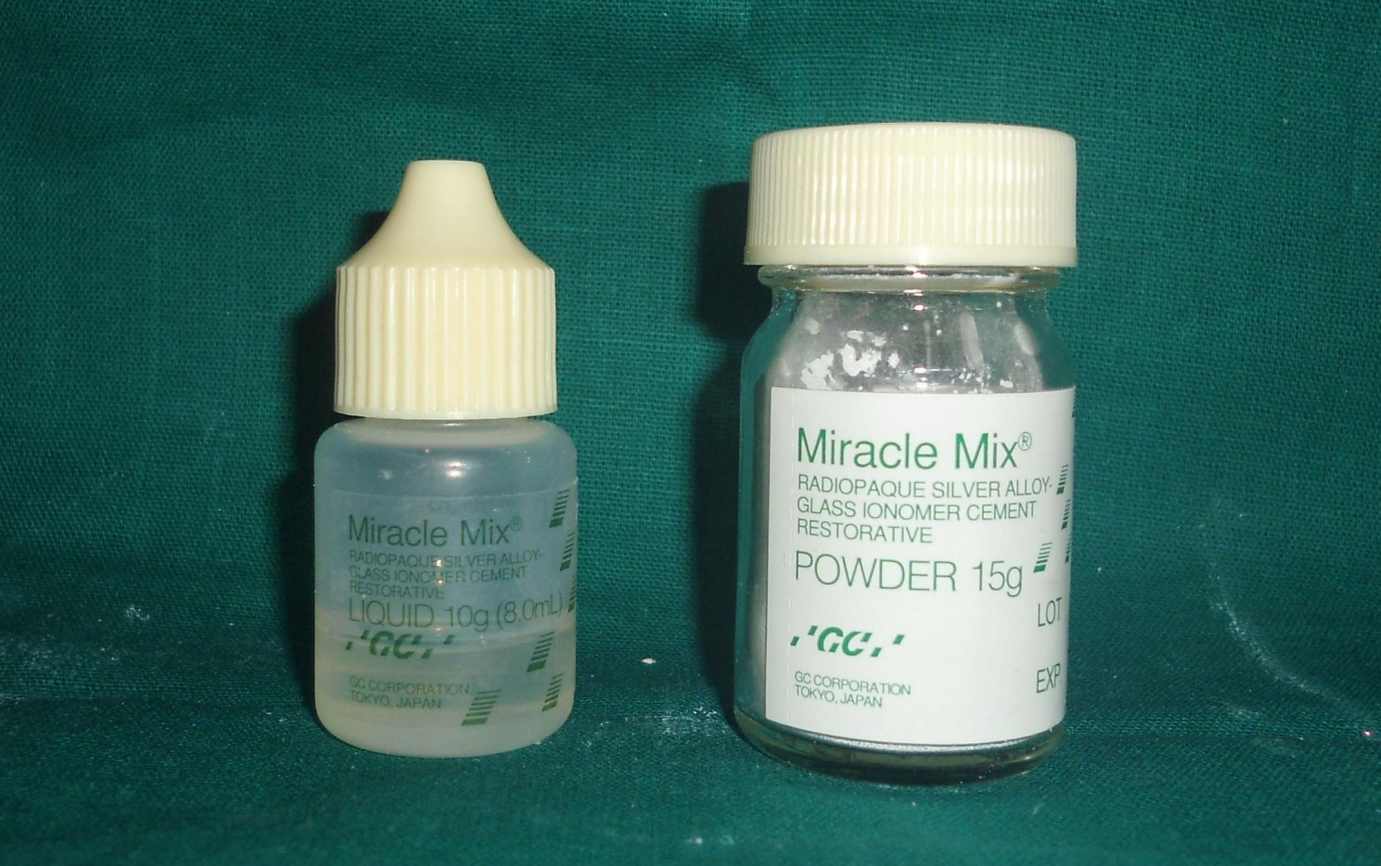
Miracle Mix (GC Fuji Miracle Mix, Japan)

Teeth restored with Miracle Mix [GC Fuji Miracle Mix, Japan. Batch No – 1410021 (Figure 2)]

Group III

**Figure 3:**
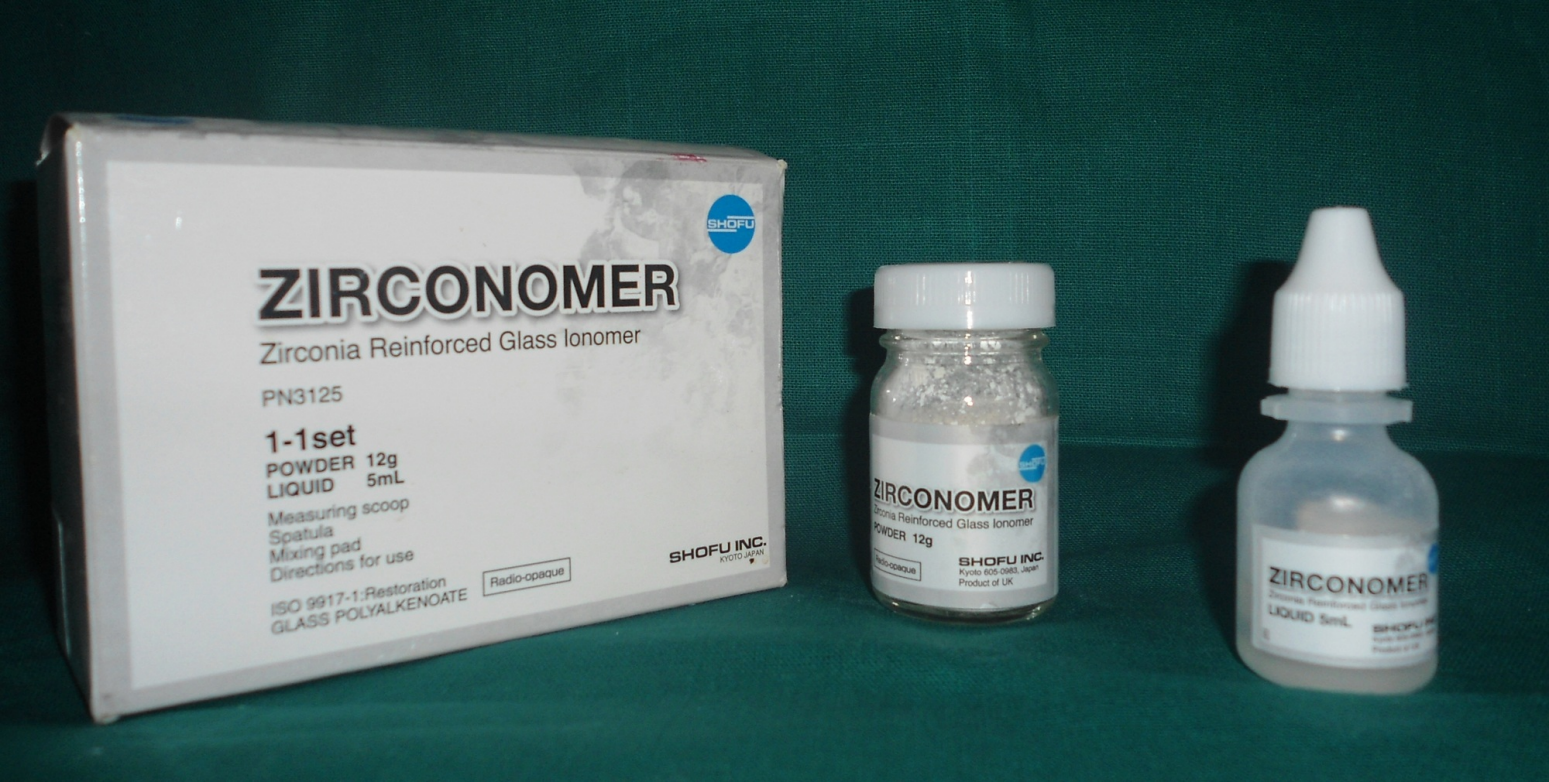
Zirconomer (Zirconia-infused glass ionomer cement – SHOFU, Japan)

Teeth restored with Zirconomer [Shofu Inc., Japan. Batch No – 071415-5 (Figure 3)]

**Figure 4:**
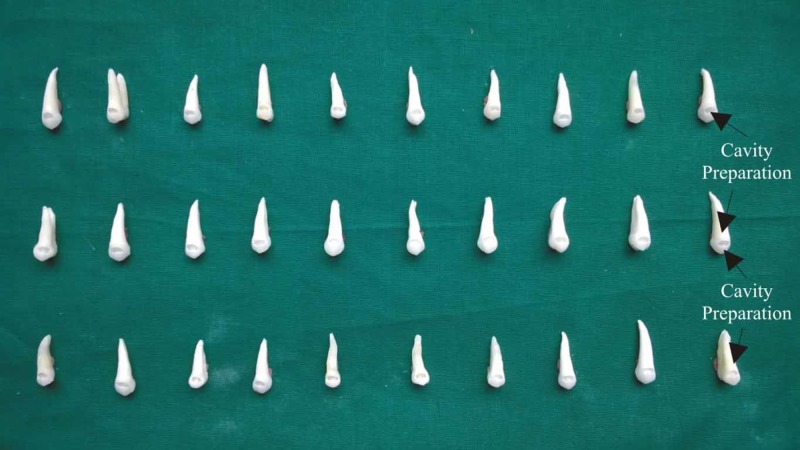
Class V cavity on premolars

Standard Class V cavities were prepared on the buccal surface of all 30 premolars using #245 carbide burs (SS White Burs Inc., New Jersey, USA) in a high speed hand-piece with a profuse volume of water coolant. The dimensions of the cavities prepared were limited to the following: mesiodistally 3 mm wide, occlusogingival height of 2 mm and depth of 2 mm (Figure 4). The dimensions of the cavities were millimetrically standardized using a Williams probe. All the preparations were performed by the same operator.

**Figure 5:**
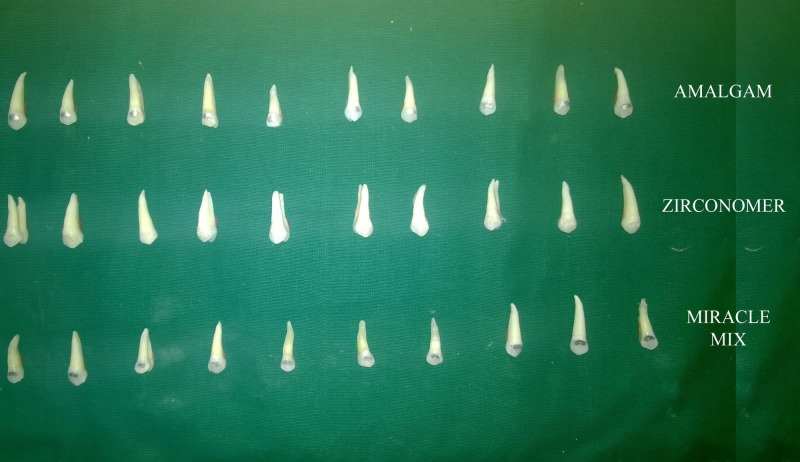
Restoration on premolars

**Figure 6:**
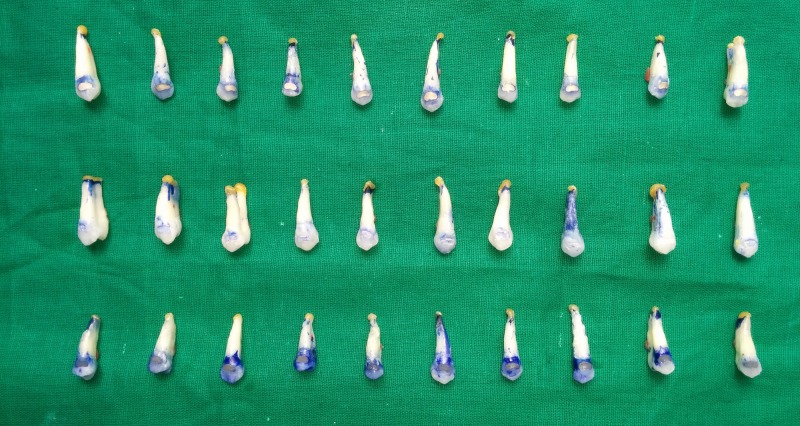
Dye penetration

The teeth were restored according to manufacturer’s instructions (Figure 5). The specimens were then placed in distilled water for 24 hours. The specimens were submitted to a thermocycling regimen 500 times between 12±20 C and 65±20 C, with a dwell time of 30 s at each temperature and 10 s intervals between the baths. Following thermocycling, the teeth apex were covered with yellow sticky wax to occlude the opening. The entire tooth surface was painted with two coats of air resistant varnish up to 1 mm of the margins of restoration. The teeth were immersed in a solution of 2% methylene blue dye for 24 hours at room temperature. Following dye penetration, the teeth were rinsed to clean the surplus stains (Figure 6).

**Figure 7:**
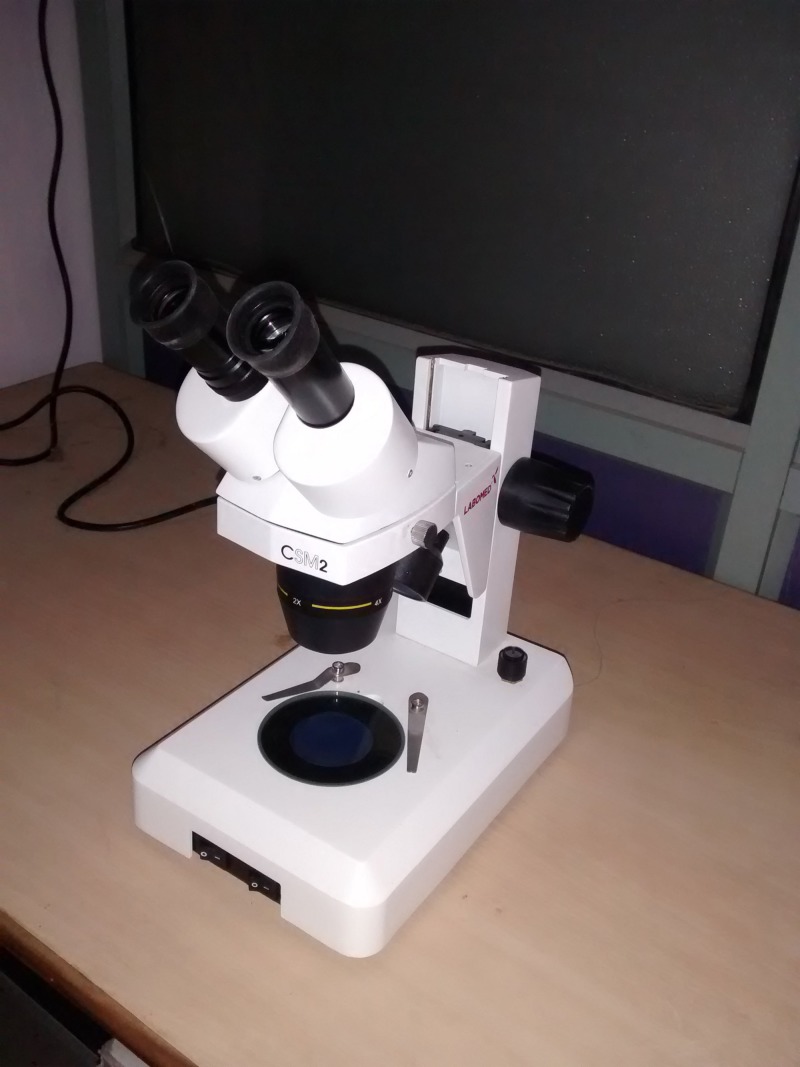
Stereo microscope (CSM-2, Labomed Inc., Los Angeles, CA, USA)

All teeth were bisected buccolingually in a longitudinal path using a diamond disc at slow speed and observed under a stereo microscope (CSM-2, Labomed Inc., CA, USA) at 40X magnification for evidence of dye penetration (Figure 7). The maximum degree of dye penetration was noted for individual specimen, and dye penetration was scored on a nonparametric scale from zero to three based on the Jessudas et al. [9]. criteria for microleakage analysis.

The following scoring criteria were used to evaluate the level of dye penetration at the tooth restoration interface by the criteria described by Jessudas et al. [9].

Score 0: No evidence of dye penetration.

Score 1: Dye penetrates to less than half the cavity depth.

Score 2: Dye penetration to full cavity depth.

Score 3: Dye penetration to axial wall and beyond.

**Figure 8:**
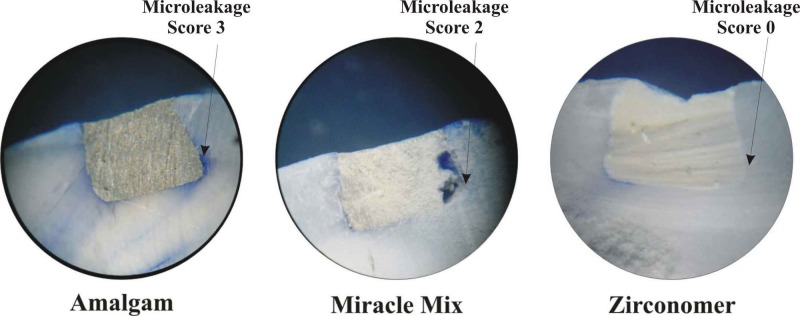
Microleakage in various materials

Microleakage was further assessed under a stereo microscope (Figure 8) by an uninvolved examiner , who was unaware of the materials used, and assessment was done according to the scale specified. Data was subjected to statistical analysis to compare the microleakage around the amalgam, Miracle mix, and Zirconomer. For each group the values of mean and standard deviations were calculated. To evaluate whether the microleakage in the three groups is homogeneous, analysis of variance was performed. Significant difference in the intergroup comparison of microleakage was evaluated by the Kruskal-Wallis test with p < 0.01 considered as significant.

The microleakage scores of the three groups are given in Table 1. Data entry and analysis were performed using Statistical Package for the Social Sciences (SPSS) version 22 (SPSS, Inc., IL, USA). The data were presented as means and standard deviations (Table 2).

**Table 1:** Scores for microleakage under stereo microscope

Specimen No.	Amalgam (Group I)	Miracle Mix (Group II)	Zirconomer (Group III)
1.	2	2	1
2.	3	2	0
3.	2	2	1
4.	3	3	2
5.	3	2	1
6.	3	2	1
7.	2	2	1
8.	3	2	2
9.	3	3	2
10.	2	3	1

**Table 2:** Descriptive statistics of microleakage of amalgam, Miracle Mix, and Zirconomer

Material	N	Mean ± Std. Deviation
Amalgam	10	2.60 ± 0.51
Miracle Mix	10	2.30 ± 0.48
Zirconomer	10	1.40 ± 0.51

The mean values of microleakage for the three groups were compared with repeated measure analysis of variance – ANOVA. By comparing the microleakage of various groups by ANOVA, a statistically significant difference was observed between the groups compared. Intergroup comparison was done further using the Kruskal-Wallis test.

## Results

A zero score was observed in one specimen in the Zirconomer group. Zirconomer showed the best result with lowest dye penetration and hence least microleakage in Class V cavity restorations. We found that 60% in the Zirconomer group showed dye penetration to less than half the cavity depth; 30% in the Zirconomer group, 70% in the Miracle Mix group, and 40% in the amalgam group showed dye penetration to full cavity depth; and 60% in the amalgam group and 30% in the Miracle Mix group showed dye penetration to the axial wall and beyond. The results of the Kruskal-Wallis test (Table 3) showed a statistically significant difference in microleakage of Group I and Group III (p = 0.001) and Group II and Group III (p = 0.002), but significant difference was not perceived between Group I and Group II (p = 0.189).

**Table 3:** Intergroup comparison of mean microleakage value dF - degrees of freedom

No.	Group	Chi-square value	dF	Significance
1	Amalgam – Miracle Mix	1.72	1	0.189
2	Miracle Mix – Zirconomer	9.197	1	0.002
3	Amalgam – Zirconomer	14.997	1	0.001
p< 0.01 was considered as significant.				

## Discussion

Ideas of restorative dentistry have transformed from “extension for prevention” to the recent attitude of “restriction with conviction”. In this era of adhesive dentistry, interfacial bonding is considered to be of paramount importance [1]. Maintaining integral marginal seal is imperative for an effective restoration. Microleakage is used as a measure to evaluate the performance of the restorative materials [3,10]. This in vitro study was carried out to evaluate and compare the microleakage of a new eco-friendly white amalgam, Zirconomer, with Miracle Mix and amalgam.

After restoration of the cavities, all the teeth were subjected to thermocycling to mimic the oral environment. Dyes can detect leakage where bacteria cannot penetrate [9,11,12]. Methylene blue was used as the dye since it can diffuse easily through the interface and is easily detectable. The main advantage is that it is not absorbed by dentinal matrix apatite crystals. It also penetrates the voids better than isotopes and has low molecular weight and thus has high penetrability.

In the present study, microleakage was evident to some extent in all groups. As suggested by Gladys et al., microleakage can be expected with all the restorative materials established till date. In the present study, we found that the least microleakage occurred in the Zirconomer group and the maximum microleakage was seen in the amalgam group [2]. The reason for this high frequency of microleakage in amalgam is likely to be the variance in the coefficient of thermal expansion between enamel and amalgam. Following thermocycling, gaps are formed between the amalgam and the enamel [13]. The poor interfacial bonding between the matrix of the Miracle Mix and AgSn amalgam alloy is of great concern for clinical application. Moreover, the color of Miracle Mix is gray, which is not aesthetically pleasing.

The Zirconomer group showed the least microleakage. Zirconomer has the virtuous attributes of good chemical and dimensional stability, toughness, and mechanical strength. Moreover it is a tooth-colored material. The wide distribution of Zirconia fillers allows a high packing density of the powder with the hydrogel salt matrix [6].

Upadhyay et al. [10] showed that nano-ionomer containing silica and zirconia fillers revealed the least microleakage. Gorseta et al. [14] witnessed least microleakage for nano-ionomers as observed with conventional glass ionomer cements and accentuated the efficacy of nano-ionomer cements. Wadenya et al. [15] recommended the use of nano-ionomer cements in atraumatic restorative techniques as there was insignificant difference between its use as an atraumatic restorative technique and as a conventional technique.

A similar study conducted by Patel et al. [11] used extracted molars with Class I restorations to evaluate and compare microleakage with the dye penetration method. They concluded that the Zirconomer group exhibited maximum microleakage when compared with the amalgam group, which was contrary to our study [11]. It must be taken into consideration that diverse study protocols such as tooth used, type of cavity preparation, and storage time may affect the results of the study.

## Conclusions

Zirconomer currently is projected as the "White Amalgam". None of the three materials were free from microleakage. Zirconia-modified glass ionomer cement demonstrated the least microleakage and proved to be better than the amalgam or Miracle Mix. In addition, improved reinforced glass ionomer cement may prove to be the ideal material in minimally invasive dentistry. However, further in vitro and in vivo studies should be performed to investigate other physical qualities of the material, and long term clinical experiences may be suggested.
